# Matrix to predict rapid radiographic progression of early rheumatoid arthritis patients from the community treated with methotrexate or leflunomide: results from the ESPOIR cohort

**DOI:** 10.1186/ar4092

**Published:** 2012-11-19

**Authors:** Bruno Fautrel, Benjamin Granger, Bernard Combe, Alain Saraux, Francis Guillemin, Xavier Le Loet

**Affiliations:** 1 Université Pierre et Marie Curie (UPMC) - Paris 6, GRC-08 EEMOIS; AP-HP Pitié Salpêtrière Hospital, Department of Rheumatology, 75013 Paris, France; 2 Montpellier I University; Department of Rheumatology, Lapeyronie Hospital, UMR 5535, 34000 Montpellier, France; 3 Brest University; Department of Rheumatology, La Cavale Blanche University Hospital, 29000 Brest, France; 4 Lorraine University, Paris-Descartes University, EA 4360 APEMAC -- Inserm, CIC-EC CIE6, CHU de Brabois, 54505 Vandoeuvre-lès-Nancy, France; 5 Rheumatology Department, Rouen University Hospital & INSERM U905, Institute for Research and Innovation in Biomedicine, Rouen University; 76031 Rouen, France

## Abstract

**Introduction:**

Early rheumatoid arthritis (RA) patients may show rapid radiographic progression (RRP) despite rapid initiation of synthetic disease-modifying anti-rheumatic drugs (DMARDs). The present study aimed to develop a matrix to predict risk of RRP despite early DMARD initiation in real life settings.

**Methods:**

The ESPOIR cohort included 813 patients from the community with early arthritis for < 6 months; 370 patients had early RA and had received methotrexate or leflunomide during the first year of follow-up. RRP was defined as an increase in the van der Heijde-modified Sharp score (vSHS) ≥ 5 points at 1 year. Determinants of RRP were examined first by bivariate analysis, then multivariate stepwise logistic regression analysis. A visual matrix model was then developed to predict RRP in terms of patient baseline characteristics.

**Results:**

We analyzed data for 370 patients. The mean Disease Activity Score in 28 joints was 5.4 ± 1.2, 18.1% of patients had typical RA erosion on radiographs and 86.4% satisfied the 2010 criteria of the American College of Rheumatology/European League Against Rheumatism. During the first year, mean change in vSHS was 1.6 ± 5.5, and 41 patients (11.1%) showed RRP. A multivariate logistic regression model enabled the development of a matrix predicting RRP in terms of baseline swollen joint count, C-reactive protein level, anti-citrullinated peptide antibodies status, and erosions seen on radiography for patients with early RA who received DMARDs.

**Conclusions:**

The ESPOIR matrix may be a useful clinical practice tool to identify patients with early RA at high risk of RRP despite early DMARD initiation.

## Introduction

The care of rheumatoid arthritis (RA) has profoundly evolved during the last decade because of new drug therapies and the early treatment paradigm. RA requires rapid referral to a rheumatologist [1-3] and early initiation of disease modifying anti-rheumatic drugs (DMARDs) to prevent long-term disease consequences, such as diabetes or hypertension [4,5]. At the same time, regular adaptation of DMARDs depending on disease activity - that is, RA tight control - has become an additional standard in RA management to achieve at least low disease activity and, if possible, disease remission [3,6-12]. Implementation of these recommendations in practice has led to better clinical outcomes [6,8,13].

The choice of the first DMARD has been the topic of many trials and guidelines. Methotrexate (MTX) has been recommended as the anchor drug because it allows for step-up strategies, that is, the addition of other synthetic or biological DMARDs if adequate response is not achieved with MTX monotherapy [3,9,14]. Leflunomide is the alternate choice because both drugs seem to have equivalent symptomatic and structural efficacy [15]. A number of trials have assessed the potential benefit of biologic DMARDs as first-line treatment for early RA [16-18]. These intensive options have been considered more efficacious than MTX in trials with a static therapeutic strategy during the first year [18]. However, in trials adopting dynamic step-up strategies, the overall benefit of biologics as a first-line agent remained questionable [6,11,12,19-21]. In addition, several economic evaluations reported that incremental cost-effectiveness ratios of biologics as first-line treatment for early RA are usually high and largely overtake the generally accepted thresholds [22,23]. These conclusions reinforced the position of MTX (or leflunomide) as the optimal first-line agent for early RA.

Despite current recommendations, for some patients, MTX may be suboptimal as first-line therapy. Several trials have shown substantial structural progression even with MTX started rapidly after disease onset. This situation has led to the development of the concept of rapid radiographic progression (RRP), defined as structural damage progression of at least five points of the van der Heijde-modified Sharp score (vSHS); the cut-off of five points corresponds to the destruction of one small joint and to the usually reported smallest detectable difference (SDD) [24-26]. The rationale for this threshold has been confirmed in two different studies. In the BeSt trial, patients with RRP during the first year of follow-up showed increased functional limitations and structural damage progression over eight years of follow-up, despite a tight control-based therapeutic strategy [27]. These results are consistent with another study of the ESPOIR cohort in which patients with RRP during the first year in the cohort, with a definition slightly different from the previous one, showed increased structural damage progression during the second and third years in the cohort [28].

The poor prognostic value associated with RRP is important and may be addressed by the development of prediction matrices to quantify the risk of RRP at one year in terms of baseline data. Such matrices are a tool than can identify patients with early RA who will show inadequate response to MTX or its equivalent [24,29-31]. These matrices have been developed in randomized clinical trials of patients with aggressive disease, who are not generally representative of patients with early RA. Therefore, the use of these matrices is limited in clinical practice, as was recently shown in the BRASS cohort [32].

Thus, we conducted a study of data for the ESPOIR cohort, which enrolled patients with early arthritis from the community (with or without unfavorable prognostic factors). We aimed to develop a prediction algorithm and matrix to identify patients with early RA at risk of RRP despite early synthetic DMARD initiation.

## Materials and methods

### Patients

Between December 2002 and March 2005, 813 patients with possible RA who were referred by rheumatologists and general practitioners to one of 14 regional centers were included in the ESPOIR cohort [33]. Inclusion criteria were age 18 to 70 years, more than two swollen joints for > 6 weeks and < 6 months, suspected or confirmed diagnosis of RA, and not taking any DMARDs or steroids except for < 2 weeks before enrollment. During the first year, patients were followed every six months. Clinical and biological data collected were disease activity by the Disease Activity Score in 28 joints-4 variables (DAS28(ESR)-4v) [34] and functional ability by the Health Assessment Questionnaire (HAQ) [35]. Radiographs of the hand and feet (antero-posterior views) were obtained at each time, as was information on therapeutic regimen. Treatment strategies were not protocol-based in the ESPOIR cohort, and patients received usual care by their rheumatologist. The protocol of the ESPOIR Cohort study was approved by the ethics committee of Montpellier University Hospital, France. All patients gave their signed informed consent to participate in the cohort.

The current study involved data for ESPOIR patients with an RA diagnosis according to their rheumatologist and initiation of a first synthetic DMARD such as MTX or leflunomide with demonstrated structural efficacy for at least three months during the first year of follow-up in the cohort.

### Structural damage assessment

X-ray data were collected in the radiography coordinating center and were read pair-wise by a well-trained investigator blinded to clinical evaluation (intra-class correlation coefficient 0.99, SDD 0.966) [4]. Structural damage was assessed qualitatively by the presence of typical RA erosions, based on their location and aspect, and quantified according to the vSHS [36,37]. RRP was defined as change in vSHS (ΔvSHS) ≥ 5 at 1 year [24,27,30,38].

### Data management and statistical analysis

Quantitative variables are expressed as mean ± SD and median. Qualitative data were expressed as number (%). The predictors of ΔvSHS were selected by a conventional two-step procedure.

The Mann-Whitney U test (for numerical data) and Fisher's exact test (for categorical data) were used in a univariate analysis to establish the statistical significance of the relation between candidate predictors and RRP, without any *a priori *assumptions about the distribution of the variables. All variables with *P *≤ 0.1 were selected for multivariate regression analysis. To construct the prediction matrix, quantitative variables selected in univariate analysis were categorized, the optimal threshold being selected on the basis of the variable distribution.

The multivariate analysis was based on a logistic regression model with a conventional backward stepwise procedure validated by a forward stepwise procedure whereby variables were optimized by the Akaike information criteria, with *P *< 0.05. The overall discrimination power of the model was evaluated by receiver operating characteristic curve (ROC) analysis and the calculation of the area under the ROC (AUC). The fit of the model was assessed by the Hosmer-Lemeshow test.

### Matrix elaboration

The RRP prediction matrix was developed by use of the model equation estimating the probability for one patient with early RA to display RRP at one year for each combination of identified predictors. The 95% confidence intervals of individual probabilities were calculated with 1,000 bootstrap replications, after removal of the outliers.

All tests involved use of R 2.12.1 (R Foundation, Vienna, Austria).

## Results

### Patient characteristics

From the ESPOIR cohort, 370 patients (45.5%) had started MTX (*n *= 335, mean dose 17.5 mg/week) or leflunomide (*n *= 35, mean dose 20 mg/week). These patients, referred to as 'synthetic DMARD (SD)-treated patients', were comparable to the rest of the cohort in terms of age, sex, swollen and tender joints, and functional limitation. However, they had shorter disease duration, higher biological inflammation values and more frequently were positive for rheumatoid factor (RF) or anti-citrullinated peptide antibodies (ACPA), had joint damage and satisfied the 2010 criteria of the American College of Rheumatology/European League Against Rheumatology (EULAR; Table 1). This indicates that the selected patients were more likely to have RA than those who did not receive one of the two drugs, illustrating a channeling bias.

**Table 1:** Baseline characteristics of the ESPOIR patients who received methotrexate or its equivalent as a first-line biologic agent for rheumatoid arthritis.

	SD-treated patients (number = 370)	All ESPOIR patients (number = 813)
Age, years	49.4 ± 11.4 (51.5)	48.1 ± 12.5 (50.1)
Female sex	271 (73.2%)	624 (76.7%)
Disease duration, weeks	15.2 ± 15.4 (57.7)	31.6 ± 37.1 (21.3)
Swollen joint count in 28 joints	7.9 ± 5.4 (7)	7.2 ± 5.4 (6)
Tender joint count in 28 joints	8.7 ± 6.9 (7)	8.4 ± 7 (6)
ESR, mm/1^st ^hour	32.7 ± 25 (26)	29.4 ± 24.6 (22)
CRP, mg/L	24.8 ± 37.7 (11)	20.3 ± 32.4 (9)
DAS28(ESR)-4v	5.4 ± 1.2 (5.2)	5.1 ± 1.3 (5.1)
IgM RF positivity	204 (55.1%)	376 (45.8%)
ACPA positivity	185 (50%)	315 (38.8%)
Typical erosion on radiographs	66 (17.8%)	100 (13.6%)
vSHS score	6.02 ± 9.7 [54]	3.71 ± 5.71 [54]
ACR/EULAR 2010 criteria	316 (85.4%)	582 (79.1%)
HAQ score	1.03 ± 0.7 (1)	0.979 ± 0.684
First-line agent:		
- No DMARDs	n.a.	207 (25.5%)
- DMARDs without structural effect	n.a.	117 (14.4%)
- Methotrexate or leflunomide	370 (100%)	396 (48.7%)
- Other DMARDs with structural effect	n.a.	56 (6.9%)
- Tumor necrosis factor blockers alone or in combination	n.a.	37 (4.6%)

Data are mean ± SD (median) or number (%); n.a.; not available. Baseline CRP level (normally < 10 mg/l), IgM and IgA RF (ELISA, Menarini, France; positive > 9 UI/ml) and anti-CCP2 antibodies (ACPA; ELISA, DiaSorin, France; positive > 50 U/ml) were detected in all patients with the same technique in a central lab (Paris-Bichat). ACPA, anti-citrullinated protein antibody; ACR/EULAR, American College of Rheumatology/European League Against Rheumatism; CRP, C-reactive protein; DAS28(ESR)-4v, Disease Activity Score in 28 joints-4 variables, using erythrocyte sedimentation rate; DMARD, disease-modifying anti-rheumatic drugs; ESR, erythrocyte sedimentation rate; HAQ, Health Assessment Questionnaire; IgM, immunoglobulin M; RF, rheumatoid factor; SD, synthetic DMARD; vSHS, van der Heijde-modified Sharp score.

Patients who initially received MTX started the drug on average 27.4 ± 15.4 weeks after disease onset. Among them, 302 were still receiving MTX at 1 year, 19 had switched to another SD and 11 to a biologic agent, mainly TNF blockers (Table 2). Among patients receiving leflunomide, only two switched to a biologic agent at one year.

**Table 2:** Evolution of DMARD treatment and disease during the first year for ESPOIR patients with early rheumatoid arthritis (RA).

First DMARD			Treatment at 1 year			
**Type**	**N**	**Delay between RA onset and DMARD start (weeks)^a^**		**N**	**DAS28(ESR)-4v at baseline**	**DAS28(ESR)-4v at 1 year**

Methotrexate^b^	335	26.7 ± 11.6 (24.3)	Methotrexate	302	5.3 ± 1.3 (5.2)	3.2 ± 2.7 (3.0)
		35 ± 15.3 (39.4)	Other synthetic DMARD(s)^c^	19	5.3 ± 1.4 (5.3)	4.1 ± 1.5 (4.1)
		30.5 ± 0.7 (21.6)	Biologic agent^d^	11	5.7 ± 0.4 (5.5)	3.9 ± 0.9 (4.2)
Leflunomide	35	48.9 ± 11.6 (46.9)	Methotrexate	9	5.8 ± 1.3 (5.6)	4.0 ± 2.7 (2.9)
		22.5 ± 15.3 (20.7)	Other synthetic DMARD(s)	24	5.4 ± 1.4 (5.5)	3.3 ± 1.5 (3.0)
		37.8 ± 0.7 (37.8)	Biologic agent^d^	2	5.9 ± 0.4 (5.9)	4.2 ± 0.9 (4.2)

Data are mean ± SD (median). ^a^Delay between RA onset and DMARD start (weeks) (*P *= 0.479): Methotrexate: 27.2 ± 15.1 (24.7); Leflunomide: 30.1 ± 18.1 (24.6); ^b^at one year: one patient did not receive any DMARDs and data for two patients were not available; ^c^leflunomide or salazopyrine; ^d^adalinumab, etanercept, infliximab or anakinra. DAS28(ESR)-4v, Disease Activity Score in 28 joints-4 variables, using erythrocyte sedimentation rate; DMARD, disease-modifying anti-rheumatic drugs; N, number.

### Structural damage

For the SD-treated patients, the mean structural progression within the first year in terms of vSHS was 1.6 ± 5.5 (median 0), mainly because of progression of joint erosion (Table 3, Additional file 1-Figure S1). The progression occurred in 126 (34.1%) patients (ΔvSHS ≥ SDD) with mean vSHS change of 5.4 ± 7.0 (median 2). Among them, 41 had RRP, which represented 11.1% of all SD-treated patients and 32.2% of patients with disease progression.

**Table 3:** Structural disease progression in patients with early RA who received DMARDs and the entire ESPOIR cohort.

	SD-treated patients	All ESPOIR patients
**All patients**	(number = 370)	(number = 736^a^)
- ΔvSHS total	1.6 ± 5.5 (0)	0.96 ± 4.4 (0)
- ΔvSHS erosion	1.5 ± 4.7 (0)	0.96 ± 3.8 (0)
- ΔvSHS narrowing	0.2 ± 1.8 (0)	0.01 ± 1.81 (0)
**Patients with structural progression^b^**	(number = 126, 34.1%)	(number = 187, 25.4%)
- ΔvSHS total	5.4 ± 7.0 [54]	5.0 ± 6.3 [54]
- ΔvSHS ≥ 5 points (RRP)	41 (11.1%)	58 (7.9%)

Data are mean ± SD (median) or number (%).^a^X-ray data were available for only 736 patients; ^b^Structural progression was defined as ΔvSHS ≥ 1 point (that is, the smallest detectable difference). DMARD, disease-modifying anti-rheumatic drugs; RRP, rapid radiographic progression; SD, synthetic DMARD; ΔvSHS, change in van der Heijde-modified Sharp score.

### RRP determinants

The variables associated with RRP on univariate analysis were disease duration, swollen joint count (SJC) and CRP level (only when considered a categorical variable), RF and ACPA status, alone or combined, and at least one typical erosion seen on hand or foot radiographs (Table 4). Of note, there were no differences between the two treatment populations (MTX or leflunomide) with regard to these determinants. The final multivariate model included only SJC and CRP level (with two different thresholds for each), as well as combined RF or ACPA status and presence of typical RA erosions, which were predictors of RRP (Table 5). ROC analysis confirmed the good discriminating power of the model with an area under the curve (AOC) of 0.754 [see Additional file 1, Figure S2].

**Table 4:** Association of main baseline characteristics of patients with early RA and rapid radiographic progression (RRP) of rheumatoid arthritis (univariate analysis).

	With RRP (number = 41)	Without RRP (number = 329)	*P *value^a^
Age, years	49.8 ± 12 (52.7)	49.3 ± 11.5 (7)	0.6
Sex	31 (75.6%)	240 (73%)	0.85
Disease duration, weeks	18.6 ± 8.4 (18.6)	14.7 ± 8.1 (13)	0.007
SJC in 28 joints	8.6 ± 6.1 (8)	7.8 ± 5.3 (7)	0.5
< 14	31 (75.6)	285 (86.6)	
14 to 20	6 (14.6)	32 (9.7)	0.08
≥ 20	4 (12.2)	12 (3.6)	
TJC in 28 joints	8.3 ± 6.3 (7)	8.7 ± 6.9 (7)	0.97
ESR, mm/1 hr	32.6 ± 21.3 (30)	32.7 ± 25.4 (25)	0.5
CRP, mg/L	26.2 ± 27.9 (14)	24.6 ± 38.7 (11)	0.12
< 4	4 (9.7)	89 (27.1)	
4 to 35	25 (61)	168 (51.1)	0.04
≥ 35	12 (29.3.8)	72 (21.9)	
Elevated ESR or CRP level	38 (90.3)	289 (87.8)	0.45
DAS28(ESR)-4v	5.3 ± 1.2 (5.4)	5.3 ± 1.2 (5.2)	0.8
RF positivity	29 (70.7)	175 (53.2)	0.04
ACPA positivity	31 (75.6)	154 (46.8)	0.0008
RF or ACPA positivity	32 (78)	190 (57.8)	0.01
HAQ score	0.95 ± 0.6 (1)	1.04 ± 0.7 (1)	0.5
Typical RA erosion	18 (44)	48 (14.6)	< 0.0001
Prednisone ≥ 7.5 mg/d	2 (4.9)	35 (10.6)	0.4
≥ 5 mg/d	7 (17.7)	91 (27.7)	0.19
Delay before 1st DMARD initiation ≥ 6 months after RA onset	22 (53.7)	147 (44.7)	0.32
Satisfaction of 2010 ACR/EULAR criteria	38 (92.7)	278 (84.5)	0.24

Data are mean ± SD (median) or number (%). RRP-positive status was defined by progression of the vSHS total score ≥ 5 points between baseline and one year (of note, among the 46 patients with RRP, 44 had progression with vSHS erosion score ≥ 5 points and 13 with vSHS narrowing score ≥ 5 points). ^a^Student *t *test or Mann-Whitney U test for continuous variables and Fischer exact test for categorical variables, with *P *< 0.1. ACPA, anti-citrullinated protein antibody; ACR/EULAR, American College of Rheumatology/European League Against Rheumatism; CRP, C-reactive protein; DAS28(ESR)-4v, Disease Activity Score in 28 joints-4 variables, using erythrocyte sedimentation rate; DMARD, disease-modifying anti-rheumatic drugs; ESR, erythrocyte sedimentation rate; HAQ, Health Assessment Questionnaire; RF, rheumatoid factor; SJC, swollen joint count; TJC, tender joint count; vSHS, van der Heijde-modified Sharp score.

**Table 5:** Determinants of rapid radiographic progression of RA for patients with early RA who received DMARDs (multivariate analysis).

	Estimate	Standard Error	z value	*P *value
Swollen joint count 14 to 20	0.27	0.53	0.52	0.60
Swollen joint count ≥ 20	1.25	0.68	1.84	0.06
CRP 4 to 35 mg/L	0.83	0.57	1.45	0.15
CRP ≥ 35 mg/L	0.86	0.63	1.36	0.17
ACPA status	1.11	0.40	2.75	0.006
Typical RA erosion	1.31	0.63	3.53	0.0004
Intercept	-3.94	0.58	-6.74	1.62e-11

ACPA, anti-citrullinated protein antibody; CRP, C-reactive protein; DMARD, disease-modifying anti-rheumatic drugs; RA, rheumatoid arthritis

### Matrix elaboration

**Figure 1:**
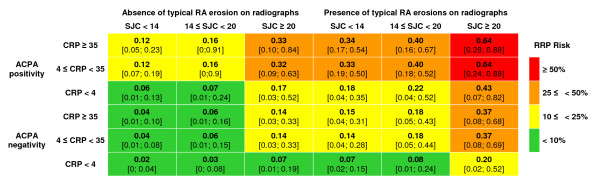
**ESPOIR prediction matrix**. ESPOIR prediction matrix for use in daily practice in assessing the risk of rapid radiographic progression (RRP; change in vSHS ≥ 5 points at 1 year) in patients with early rheumatoid arthritis in terms of baseline characteristics. ACPA, anti-citrullinated protein antibody; CRP, C-reactive protein (mg/L); vSHS, van der Heijde-modified Sharp score.

From these data, we developed a risk matrix (Figure 1, Additional file 1, Figure S3) that indicates the probability of RRP for patient profiles in terms of baseline SJC, CRP level, ACPA status, and erosions seen on radiography. For example, for a patient without typical RA erosions on radiographs, no ACPA positivity, who receives MTX, the probability of RRP at one year is 2% with SJC < 14 and CRP level < 4 mg/L but 14% with SJC ≥ 20 and CRP level ≥ 35 mg/L. However, for a patient with typical RA erosions on radiographs, ACPA positivity, who receives MTX, the probability of RRP is 18% with SJC < 14 and CRP level < 4 mg/L but 64% with SJC ≥ 20 and CRP level ≥ 35 mg/L. In terms of the high RRP probability, treatment for the latter patients with only MTX or equivalent could be considered suboptimal. Such patients represented 0.5% (*n *= 2) of the ESPOIR population if the 50%-threshold is used (red boxes) and up to 12.4% (*n *= 46) if the 25%-threshold is used (red and orange boxes).

## Discussion

The present study allowed for the construction of a matrix to predict the risk of RRP for patients with early RA despite MTX or leflunomide therapy. Since RA is considered a medical emergency [39] requiring rapid referral to a rheumatologist [1-3] and early initiation of DMARDs to prevent disease progression, the ESPOIR matrix might help rheumatologists in daily practice identify patients at high risk of SD failure, and thus make better-informed and evidence-based therapeutic decisions. The community-based nature of the study is one of the major strengths of this work, which makes patient typology more representative of daily clinical practice than that of randomized clinical trials. The ESPOIR cohort included patients with early arthritis and possible, probable or definite diagnosis of RA. However, no specific disease activity level or prognostic markers of severity were required for inclusion [33].

For several reasons, we focused on patients who had started receiving MTX or leflunomide during the observation period. First, therapeutic decisions were not protocol-based in the ESPOIR cohort, and patients received treatment according to standard care by their rheumatologist. Patients with the most active or structurally aggressive disease were more likely to receive the most effective drugs such as MTX or TNF blockers, which highlights a channeling bias [4,40]. Four gross therapeutic strategies can be distinguished: no DMARD during the first year in the cohort, DMARDs without demonstrated structural efficacy such as hydroxychloroquine, DMARDs with demonstrated structural benefit such as MTX or leflunomide, or biologics such as TNF blockers. Without any adjustment, the progression in vSHS was substantially higher in the latter two groups, which shows that patients receiving the most efficacious drugs were identified by their rheumatologists as being likely to have active and erosive RA [4]. The use of propensity scores could in theory adjust on these confounding factors. Although different models were tested, we did not achieve a satisfactory adjustment, able to make the different treatment groups comparable at baseline. Thus, the analysis was stratified and only focused on the more clinically relevant and adequately sample-sized group, that is, the group of patients treated with either MTX or leflunomide, the two most widely used therapies in early RA. Although several synthetic DMARDs have shown their ability to prevent structural damage progression, only two seem to have similar efficacy (that is, MTX and leflunomide) in a recent systematic literature review conducted under the auspices of EULAR [15]. Therefore, sulfasalazine, gold salts or other synthetic DMARDs were excluded in the patient selection process. This choice also appeared more clinically relevant because this therapeutic option is considered optimal for most patients [17]. Our data indicate that other options, for example, biologics, could be better for the patients with high RRP risk (red boxes in Figure 1). In an explanatory analysis, the patients treated with TNF-blockers as first line agent (*n *= 37, with complete data available for only 27) were plotted in the matrix; their observed RRP risk was lower than the expected RRP risk.

Several prediction matrices have been recently proposed. Three were derived from results of randomized controlled clinical trials assessing the efficacy of TNF-blocking agents [20,24,29-31,41], so patients with early RA with substantial disease activity and/or severity were included as candidates for biologics. Moreover, in these trials, therapeutic adaptation was protocol-based - fixed strategy over one year or tight control according to a predefined scheme - which, in both cases, is not quite consistent with usual care in which treatment adaptations are often looser, thus enabling suboptimal disease control [42,43]. Only one algorithm was developed from data from an early RA cohort, SONORA, in Canada and the United States but has not been published [44]. The predictors identified to build the different matrices were partly overlapping. In the ASPIRE matrix [24], the three constitutive variables comprising the matrix were RF status, SJC and erythrocyte sedimentation rate (ESR); baseline joint damage was not included, although it is a strong predictor of further structural damage in RA [45]. In the BeSt study [30], RF or ACPA status, number of erosions at baseline and CRP level but not SJC, another marker of disease severity, were used in the risk matrix [45]. In the SWEFOT trial [20,29,31,41], the proposed matrix included smoking status, CRP level and erosions at baseline; ACPA status was not significantly associated with RRP, which is not consistent with the literature [45]. This finding may be explained in part by the confounding of the strong association of smoking status and ACPA status [46-48]. The ESPOIR matrix may appear more comprehensive than the other matrices because it included all the known RA prognostic markers. Of interest, in the observational cohort SONORA, the proposed matrix included most of the same parameters: baseline DAS28(ESR)-4v, vSHS and ACPA status [44].

Our study has some limitations. The first pertains to MTX dosage, because several doses have been proposed in the literature. Our patients received 17.5 mg/week, on average, which may be considered slightly lower than that used in other settings [49]. Another concern relates to low-dose prednisone therapy, which has been efficacious in preventing structural damage progression [50-52]. In the ESPOIR cohort, 42% of the patients received steroids during the first year of follow-up, with a mean dose of 5.5 ± 3.7 mg/d (median 5 mg/d). This daily dose was quite low, and our analyses did not reveal any association between prednisone intake and RRP.

The quality of care could not be assessed in the ESPOIR cohort, especially in terms of optimal or suboptimal timing of RA diagnosis or MTX introduction, two key elements of future disease control [3,4,13,53]. Moreover, the implementation and respect of tight control was not identifiable in the cohort and may have had an impact on structural progression [42]. However, this situation may suggest that our results are representative of clinical practice, as stated above. Another important limitation is the lack of validation of our matrix in a different population, which is a limitation in all RA matrices. Attempts have been made to cross-validate the ASPIRE, BeSt and SWEFOT matrices in the SWEFOT trial population [41] and in the BRASS cohort, an established RA cohort [32]. In both cases, the performance of the different matrices in different populations was disappointing. Whether the more comprehensive ESPOIR matrix may be optimal in different populations remains to be demonstrated, as well as the performance of the other matrices in the ESPOIR population (work in progress). Finally, all matrices have limitations inherent in their development that relate to multivariate analyses performed in limited samples of patients. The RRP risk was thus a predicted risk as opposed to an observed risk. Some of the matrix boxes were rather poorly populated and may explain the only fair performance of matrices in populations different from those in which they have been developed.

## Conclusions

The ESPOIR matrix is a novel tool developed in a real life setting to help rheumatologists in usual daily practice to identify patients with early RA at high risk of RRP despite MTX or leflunomide therapy. The performance of this tool needs to be validated in other patient populations. Then, it will become a quite relevant instrument to guide rheumatologists in their therapeutic decision making, especially to detect patients for whom MTX may not be optimal therapy.

## Abbreviations

ACPA: anti-citrullinated peptide antibodies; ACR: American College of Rheumatology; AUC: area under the curve; CRP: C-reactive protein; DMARD: disease-modifying anti-rheumatic drugs; DAS28(ESR)-4v: disease activity score in 28 joints-4variables, using erythrocyte sedimentation rate; ESR: erythrocyte sedimentation rate; EULAR: European League Against Rheumatism; HAQ: Health Assessment Questionnaire; MTX: methotrexate; RA: rheumatoid arthritis; RF: rheumatoid factor; ROC curve: receiver operating characteristic curve; RRP: rapid radiographic progression; SD: synthetic DMARD; SDD: smallest detectable difference; TNF: tumor necrosis factor; vSHS: van der Heijde-modified Sharp score; SJC: swollen joint count; TJC: tender joint count; TNF: tumor necrosis factor.

## Competing interests

The authors declare that they have no competing interests.

## Authors' contributions

The design of the study was conceived by BF, BG, BC and XL. Data collection, management and analysis were performed by BF, BC, AS, FG and XL. All authors participated in the interpretation of the results and manuscript writing. All have read and approved the final version of the manuscript for publication.

## Supplementary Material

Additional file 1 **Supplemental figures**. **Figure S1: **Cumulative plot of the vSHS score variation between baseline and month 12. **Figure S2: **Fit of the final model estimated by receiver operating characteristic curve analysis. **Figure S3: **Observed patient frequencies in the different cells of the ESPOIR matrix. Click here for file
