# Supplementary material for: Matrix to predict rapid radiographic progression of early rheumatoid arthritis patients from the community treated with methotrexate or leflunomide: results from the ESPOIR cohort
Source: Arthritis Res Ther. 2012 Nov 19;14(6):R249. doi: 10.1186/ar4092 (PMC3674616; doi:10.1186/ar4092)
Supplement: Additional file 1 — Supplemental figures. Figure S1: Cumulative plot of the vSHS score variation between baseline and month 12. Figure S2: Fit of the final model estimated by receiver operating characteristic curve analysis. Figure S3: Observed patient frequencies in the different cells of the ESPOIR matrix. [file ar4092-S1.DOC]

**Figure S1:** Cumulative plot of the vSHS score variation between baseline and month 12

**Figure S2:** Fit of the final model estimated by receiver operating characteristic curve analysis


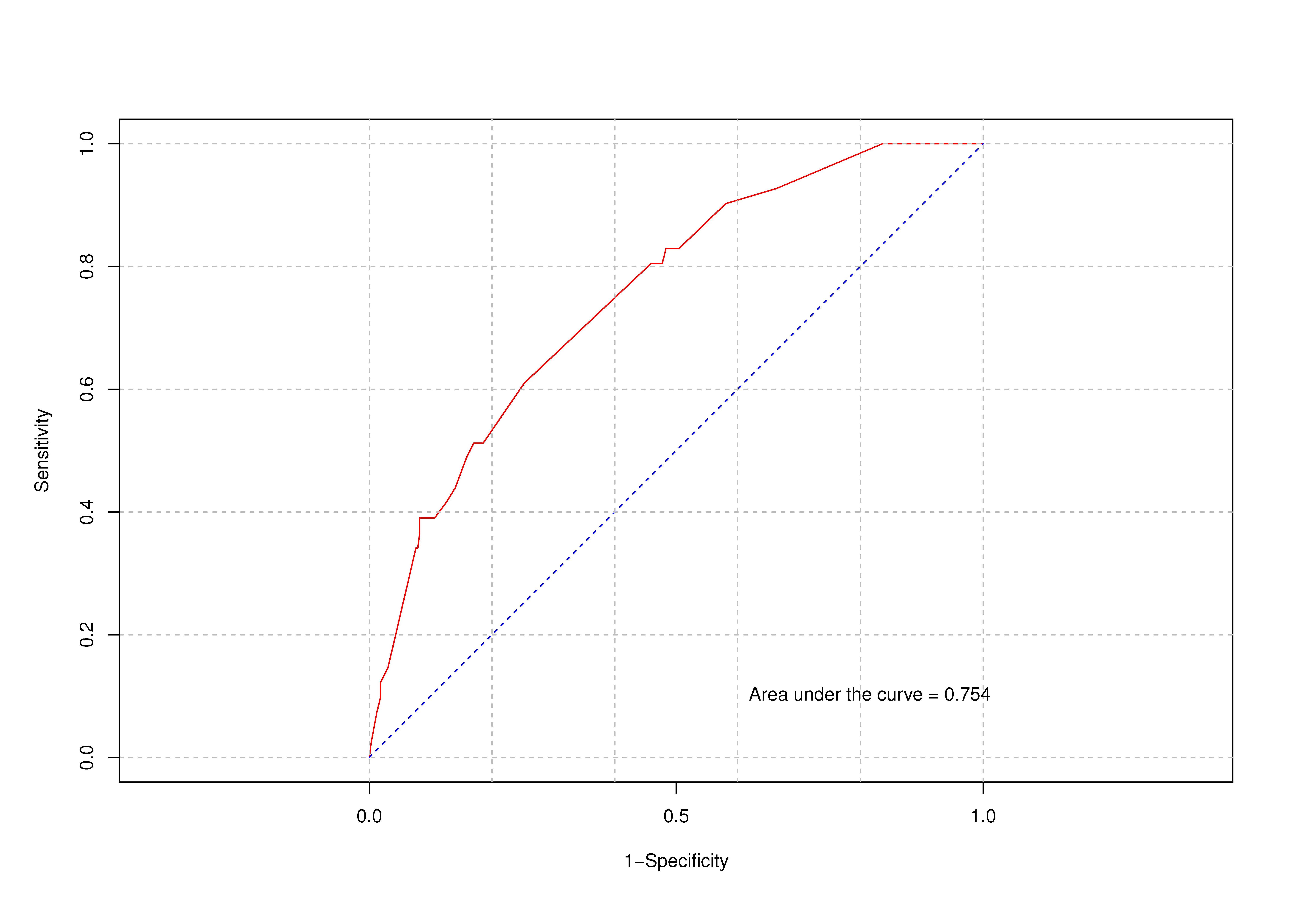


**Figure S3:** Observed patient frequencies in the different cells of the ESPOIR matrix

|  |  | **Absence of typical RA erosion on radiographs** | | | **Presence of typical RA erosions on radiographs** | | |  |  |  |
| --- | --- | --- | --- | --- | --- | --- | --- | --- | --- | --- |
|  |  | **SJC < 14** | **14 ≤ SJC < 20** | **SJC ≥ 20** | **SJC < 14** | **14 ≤ SJC < 20** | **SJC ≥ 20** |  |  |  |
| **ACPA positivity** | **CRP ≥ 35** | 34  *(29, 5)* | 3  *(3, 0)* | 2  *(1, 1)* | 8  *(5, 3)* | 6  *(4, 2)* | 2  *(1, 1)* |  | **RRP Risk** | |
|  | **4 ≤ CRP < 35** | 77  *(69, 8)* | 9  *(8, 1)* | 3  *(2, 1)* | 23  *(15, 8)* | 4  *(2, 2)* | 0  *(0, 0)* |  |  | **≥ 50%** |
|  | **CRP < 4** | 30  *(27, 3)* | 0  *(0, 0)* | 0  *(0, 0)* | 4  *(4, 0)* | 1  *(0, 1)* | 0  *(0, 0)* |  |  | **25 ≤ < 50%** |
| **ACPA negativity** | **CRP ≥ 35** | 30  *(29, 5)* | 5  *(4, 1)* | 6  *(5, 1)* | 7  *(6, 1)* | 1  *(1, 0)* | 0  *(0, 0)* |  |  | **10 ≤ < 25%** |
|  | **4 ≤ CRP < 35** | 60  *(57, 3)* | 6  *(6, 0)* | 5  *(5, 0)* | 8  *(6, 2)* | 1  *(1, 0)* | 1  *(0, 1)* |  |  | **< 10%** |
|  | **CRP < 4** | 49  *(49, 0)* | 7  *(7, 0)* | 0  *(0, 0)* | 5  *(5, 0)* | 1  *(1, 0)* | 0  *(0, 0)* |  |  |  |

Numbers presented in each cells are: total number of patients (*number of RRP- patients,* *number of RRP+ patients)*
